# Dietary determinants and public health implications of obesity in the Aseer region, Saudi Arabia

**DOI:** 10.3934/publichealth.2025058

**Published:** 2025-12-11

**Authors:** Ali Mohieldin

**Affiliations:** Department of Public Health, College of Applied Medical Sciences, King Khalid University, Abha, Saudi Arabia

**Keywords:** obesity, Saudi Arabia, Aseer region, risk factors, dietary habits

## Abstract

**Background and objectives:**

Obesity has emerged as a major public health concern in Saudi Arabia, driven by rapid lifestyle transitions and dietary changes. This study aimed to assess the prevalence and risk factors of obesity among adults in the Aseer region.

**Methods and study design:**

A cross-sectional study was conducted among 430 adults residing in the Aseer region. Data was collected through an online self-administered questionnaire, including sociodemographic factors, dietary intake, and lifestyle habits. Statistical analyses included descriptive statistics, chi-square tests, and logistic regression to examine associations with obesity (BMI ≥ 30 kg/m²).

**Results:**

Among participants, 25.3% were obese, and 48.1% were overweight. Obesity was significantly associated with female gender (73.4% of the obese group), age ≥ 36 years, and marital status (p < 0.05). Frequent consumption of sugary foods and physical inactivity also emerged as significant predictors. Logistic regression revealed female gender, being single, and a lack of physical activity as independent predictors of obesity (p < 0.05).

**Conclusions:**

Obesity prevalence in Aseer is substantial, particularly among women and older adults. Tailored interventions that promote physical activity should be prioritized, and although frequent sugary food intake was associated with obesity in bivariate analysis, this relationship did not remain statistically significant in the multivariate model and should therefore be interpreted with caution.

## 1. Introduction

Obesity has become a defining public health issue of the 21^st^ century, affecting nearly every region of the world. It is a complex, multifactorial condition associated with profound metabolic, cardiovascular, and musculoskeletal consequences, including type 2 diabetes, hypertension, dyslipidemia, ischemic heart disease, stroke, and certain cancers [1],[2]. Globally, more than 650 million adults are classified as obese, and the prevalence continues to rise, particularly in rapidly transitioning societies [3].

The Middle East and North Africa (MENA) region is at the epicenter of this epidemiological transition. Economic growth, improved living standards, and widespread urbanization have brought profound lifestyle changes that favor energy-dense diets and reduced physical activity [4],[5]. Saudi Arabia exemplifies this trend: national surveys indicate obesity prevalence rates exceeding one-quarter of the adult population, with particularly high rates among women [4],[6]. These patterns have prompted the Saudi Ministry of Health (MOH) and the Saudi Food and Drug Authority (SFDA) to prioritize obesity prevention in the National Health Transformation Program and Vision 2030 strategy, emphasizing health promotion, dietary reform, and the creation of supportive environments for physical activity [7],[8].

Dietary factors are among the most important, yet modifiable, drivers of obesity. Increased consumption of sugar-sweetened beverages, sweets, and fast foods, alongside inadequate intake of fruits, vegetables, and whole grains, contributes to excessive caloric intake and poor nutrient balance [9]. Evidence from Saudi Arabia suggests that these dietary behaviors, reinforced by high availability of processed foods and shifts from traditional diets, play a central role in rising obesity rates [10]. In parallel, sedentary lifestyles, limited access to recreational facilities, and cultural factors further amplify the risk [4],[11].

Recent Saudi data underscore the continued public health concern of obesity. For instance, a four-year surveillance study (2020–2023) reported persistently high obesity prevalence across all regions [12]. Furthermore, the 2024 Health Determinants Statistics Publication by GASTAT noted that 23.1% of adults aged 15+ are obese. Findings from the General Authority for Statistics reaffirm the importance of region-specific surveillance and intervention strategies to address subnational variation and rising trends [13].

The Aseer region in southwestern Saudi Arabia is notable for its unique geographic and cultural characteristics. While rural and highland communities have historically relied on diets rich in whole grains, legumes, and locally produced fruits and vegetables, urbanization has brought greater dependence on convenience foods and sugary products [14],[15]. Moreover, the mountainous terrain and sociocultural norms can limit opportunities for sustained physical activity, particularly among women. Despite these contextual factors, there is a paucity of research examining obesity prevalence and its dietary determinants in Aseer. Most national surveys pool data across regions, which may mask important subregional variations critical for designing targeted interventions.

Given these knowledge gaps, this study aimed to assess the prevalence of obesity and identify its sociodemographic, dietary, and behavioral predictors among adults in Aseer. By situating the findings within the broader framework of Saudi Vision 2030 and global non-communicable disease (NCD) prevention efforts, this work contributes to shaping culturally tailored interventions and informing both regional and national obesity control strategies [7],[8],[11].

## 2. Materials and methods

### 2.1. Study design and setting

This cross-sectional study was conducted in the Aseer region of southwestern Saudi Arabia, which has a population of approximately 2.2 million people [13]. Data collection was carried out between January and June 2025.

### 2.2. Study population and eligibility criteria

Participants were adults (≥18 years) who had lived in Aseer for at least six months. Exclusion criteria included pregnancy, chronic medical conditions that directly influence body weight (e.g., thyroid disorders, cancer, severe gastrointestinal disease), and incomplete questionnaires. Recruitment used digital platforms (social media, university mailing lists, community groups) to maximize reach across different age, sex, and residence categories.

### 2.3. Sample size determination

The sample size was determined using Yamane's formula [16] at a 95% confidence level and 5% margin of error. The minimum required sample was 384. To account for non-responses, a 10% surplus was added, giving a target of 422. A total of 430 valid responses were obtained, ensuring sufficient statistical power.

### 2.4. Data collection and measures

A structured, bilingual (Arabic/English) questionnaire adapted from the WHO STEPS tool [17],[18] was used. The instrument was reviewed by subject experts at King Khalid University (KKU) and piloted with 30 adults to ensure clarity and cultural appropriateness. The questionnaire covered:

Sociodemographic data: age, sex, marital status, education, occupation, household income, and place of residence.

Lifestyle behaviors: smoking status and physical activity. Physical activity was assessed in line with WHO guidelines [11], capturing both frequency and duration. For the regression, physical activity was coded as “active” (any regular activity meeting WHO recommendations) vs. “inactive”.

Dietary intake was assessed using a semi-quantitative food frequency format. For each food group, participants indicated consumption frequency using predefined categories: “rarely/never”, “1–2 times per week”, “3–4 times per week”, and “≥5 times per week”. Standard portion sizes were described (e.g., one fruit serving = one medium piece; sugary foods = one standard sweet/dessert item). These frequency cutoffs were adapted from the WHO and national SFDA dietary surveillance tools. Dietary habits were assessed as the frequency of consumption of key food groups, including fruits, vegetables, whole grains, dairy, meats, sugary foods, and sugar-sweetened beverages, using a semi-quantitative food frequency approach adapted from national surveys [7],[8].

Anthropometrics: self-reported height and weight were used to calculate body mass index (BMI, kg/m²). Participants were categorized as underweight (<18.5), normal weight (18.5–24.9), overweight (25.0–29.9), or obese (≥30.0), per WHO standards [2].

### 2.5. Ethical considerations

The study was approved by the Research Ethics Committee of King Khalid University (Approval code: ECM#2024–3190). Participants provided electronic informed consent, and anonymity was preserved throughout.

### 2.6. Statistical analysis

All analyses were conducted using IBM SPSS Statistics version 27 (IBM Corp., Armonk, NY, USA) [19]. Descriptive statistics (mean ± standard deviation for continuous variables; frequencies and percentages for categorical variables) summarized participant characteristics. Bivariate associations between obesity (BMI ≥ 30 vs. <30 kg/m²) and categorical predictors were tested with chi-square tests. Continuous variables were compared using independent-samples t-tests. All theoretically relevant variables (age, sex, marital status, education, occupation, income, smoking, residence type, physical activity, and dietary habits) were considered in a binary logistic regression model, reporting adjusted odds ratios (ORs) with 95% confidence intervals (CIs). Multicollinearity was assessed using variance inflation factors (VIF), and no evidence of problematic collinearity (VIF > 2.5) was detected. Model performance was evaluated using the Hosmer–Lemeshow goodness-of-fit test, Nagelkerke R², and classification accuracy. Statistical significance was set at p < 0.05.

## 3. Results

### 3.1. Participant characteristics

Of the 430 adults surveyed, 256 (59.5%) were female, and 174 (40.5%) were male. The mean (±SD) age of study subjects was 36.9 (±10.8) (range: 18–65) years, with 38.1% aged 36–45 years and 21.2% aged >45 years. Most participants were married (68.6%), had a university degree (73.7%), and worked in government service (52.8%). The majority resided in urban areas (84.2%) and reported a monthly household income above SAR 10,000 (55.1%). Smoking prevalence was 14.2%. The mean body mass index (BMI) of study subjects was 27.6 kg/m² (±4.3); 25.3% of participants (n = 109) were classified as obese (BMI ≥ 30), and 48.1% were overweight. Women accounted for 73.4% of the obese group. Obesity was most common among adults aged 36–45 years (41.3% of obese cases), followed by those >45 years (31.2%). See Table 1.

**Table 1.** Demographic and lifestyle characteristics of study participants (n = 430).

Variable	Categories	n (%)
Gender	Male	174 (40.5)
	Female	256 (59.5)
Age group	18–25 years	45 (10.5)
	26–35 years	130 (30.2)
	36–45 years	164 (38.1)
	>45 years	91 (21.2)
Marital status	Married	295 (68.6)
	Single	117 (27.2)
	Divorced	18 (4.2)
Education	University graduate	317 (73.7)
	High school or less	113 (26.3)
Occupation	Government employee	227 (52.8)
	Private sector	62 (14.4)
	Unemployed	104 (24.2)
	Student	24 (5.6)
Smoking status	Non-smoker	369 (85.8)
	Smoker	61 (14.2)
Residence	Urban	362 (84.2)
	Rural	68 (15.8)
Monthly income (SAR)	>10,000	237 (55.1)
	5000–10,000	144 (33.5)
	<5000	49 (11.4)

### 3.2. Bivariate associations with obesity

Bivariate analysis results are presented in Table 2. Obesity was significantly associated with age (χ² = 14.71, p = 0.002), with a higher prevalence among participants ≥36 years. Sex was significantly associated with obesity (χ² = 11.64, p = 0.001), with women more likely than men to be obese. Marital status was also associated with obesity (χ² = 7.06, p = 0.029), with obesity highest among married individuals. Sugary food intake was positively associated with obesity (χ² = 14.17, p = 0.007). Education, occupation, income, residence type, and smoking were not significantly related to obesity status (p > 0.05).

**Table 2.** Factors associated with obesity status among participants.

Variable	χ² value	df	p-value	Interpretation
Age	14.71	3	0.002	Higher prevalence in 36–45 years
Gender	11.64	1	0.001	More prevalent among females
Marital status	7.06	2	0.029	Higher in married individuals
BMI category	88.94	3	0.001	Strong BMI–obesity link
Sugary food intake	14.17	4	0.007	Higher intake linked to obesity

### 3.3. Multivariate predictors of obesity

Logistic regression analysis showed that female gender, being single, and physical inactivity were independent predictors of obesity. Although frequent sugary food intake showed a positive association in bivariate analysis, it did not reach statistical significance in the adjusted model.

The regression model demonstrated good fit (Hosmer–Lemeshow, p = 0.64), explained 48.9% of the variance in obesity status (Nagelkerke R² = 0.489), and achieved a classification accuracy of 83.7%. See Table 3.

**Table 3.** Binary logistic regression of predictors of obesity.

Predictor	p-value	OR (95% CI)	Interpretation
Gender (female)	0.001	0.126 (0.052–0.303)	Females more likely to be obese
Marital status (single)	0.031	2.968 (1.106–7.962)	Singles have increased risk
Physical activity (none)	0.011	0.344 (0.152–0.781)	Inactivity increases risk
Sugary food intake (4–5×/w)	0.120	4.644 (0.669–32.225)	Borderline predictor

Note: Adjusted odds ratios (AORs) with 95% confidence intervals are presented. The model demonstrated good fit (Hosmer–Lemeshow, p = 0.64), explained 48.9% of the variance in obesity (Nagelkerke R² = 0.489), and correctly classified 83.7% of cases.

## 4. Discussion

This study provides important insights into the epidemiology of obesity in the Aseer region of Saudi Arabia, revealing that 25.3% of adults were obese, with women and individuals aged ≥36 years disproportionately affected. These findings are in line with national data indicating that obesity is a major public health concern in Saudi Arabia and is particularly prevalent among women [20],[21]. The observed prevalence in Aseer aligns closely with national estimates (24%–28%) reported in Ministry of Health surveillance [5],[22] but also underscores potential regional variations that are often masked in national pooled analyses.

### 4.1. Gender differences in obesity

Our results demonstrate that women accounted for nearly three–quarters of obese participants, and logistic regression confirmed female gender as an independent predictor of obesity. This finding mirrors evidence from multiple national and regional studies that report higher obesity rates among Saudi women [20],[21],[23]. WHO noted that cultural norms restricting female participation in outdoor physical activities, combined with higher consumption of energy-dense foods, contribute to the gender gap in obesity prevalence across the Middle East and North Africa (MENA) region [11]. Similarly, Alqarni reported that female obesity prevalence in Saudi Arabia exceeds 30%, compared to 20% in men [10].

Interestingly, some international studies show contrasting trends. For instance, in the United States, obesity prevalence is only slightly higher in women than men (41.9% vs. 40.5%) [1], while in parts of Europe, male obesity prevalence has surpassed female rates due to changing dietary and occupational patterns [9]. These differences suggest that in Saudi Arabia, sociocultural and environmental barriers remain more influential than purely biological determinants. Therefore, interventions need to be gender-sensitive, incorporating culturally appropriate physical activity opportunities for women and community fitness programs [15],[22].

### 4.2. Age and obesity risk

The study also found that obesity was most common among adults aged 36–45 years, followed by those >45 years, consistent with the trajectory of weight gain observed in many populations [11]. These findings parallel national survey data, which reported higher obesity prevalence in middle-aged Saudis compared to younger adults [24]. Age-related metabolic changes, declining physical activity, and cumulative dietary exposures are likely to contribute to this trend.

However, some studies in the Gulf have documented increasing obesity among adolescents and young adults, highlighting the early onset of risk factors [25]. For example, Alhazmi et al. (2020) noted a growing burden of overweight and obesity among Saudi university students, with lifestyle behaviors such as irregular meal timing and fast-food consumption being major drivers [7]. While our study population was limited to adults, the age distribution suggests that preventive strategies should not only focus on older adults but also on younger age groups before weight gain becomes entrenched.

### 4.3. Marital status and obesity

One of the more novel findings of this study was that single individuals had nearly three times the odds of being obese compared to married individuals. This contrasts with prior Saudi and international studies, where marriage has frequently been associated with higher body weight due to shared eating habits, reduced physical activity, and increased social eating [26]. For example, research in Riyadh reported that married individuals had significantly higher BMI compared to single adults [25]. Although much of the older Saudi and international literature has reported higher obesity among married individuals, more recent data suggest a more complex picture. For example, a Saudi study among adults found that married participants had lower odds of overweight/obesity compared to singles [12]. In younger Saudi groups, lifestyle barriers such as limited time, low energy, and weak motivation for healthy eating and physical activity have been documented among unmarried individuals [27]. Additionally, social media–influenced dietary trends among young Saudis have been linked to higher consumption of fast food and convenience meals [28]. Given the trends toward delayed marriage in Saudi society, these behavioral patterns could increase single adults' exposure to obesogenic environments before family formation. While direct evidence linking delayed marriage to higher obesity risk in Saudi Arabia is still limited, these contextual factors may help explain the higher odds of obesity among single adults in our study.

The divergence observed in Aseer could be explained by cultural or contextual factors. Single individuals, particularly in urban areas, may rely more heavily on fast foods and sugary products due to convenience, lack of home cooking, or limited nutrition literacy [15]. Moreover, the increasing trend of delayed marriage in Saudi Arabia may prolong exposure to unhealthy dietary patterns during early adulthood. Further qualitative research is warranted to better understand these dynamics in the Aseer region.

### 4.4. Dietary risk factors

Although frequent sugary food consumption showed only a borderline significant association in the regression model, its strong bivariate association with obesity supports existing literature linking high sugar intake with weight gain and metabolic disorders. Malik et al. (2013) demonstrated a clear causal relationship between sugar-sweetened beverage intake and increased risk of obesity in longitudinal studies [29].

The lack of significant associations between obesity and other dietary variables (e.g., fruit and vegetable intake, grains, dairy) may reflect measurement limitations of self-reported food frequency data, underreporting due to social desirability, or a true absence of association in this population. Studies in other Saudi regions have documented protective effects of fruit and vegetable consumption [7],[18], suggesting that dietary patterns may vary across regions. This underlines the need for region-specific dietary surveillance and targeted nutritional education.

### 4.5. Physical inactivity

Physical inactivity emerged as a robust independent predictor of obesity in this study. Inactive participants were almost three times more likely to be obese, aligning with WHO guidelines that classify insufficient physical activity as a leading cause of global morbidity and mortality [7],[30]. Nationally, sedentary behavior remains widespread, with limited engagement in structured exercise [25]. In Aseer, geographic and cultural barriers may exacerbate inactivity, as mountainous terrain limits walkability, and gender norms constrain women's outdoor activity.

Comparable studies across the MENA region highlight physical inactivity as a major modifiable determinant of obesity [11]. For example, Alqahtani et al. (2021) observed that only 17% of Saudi adults meet WHO physical activity recommendations [25]. Our findings reaffirm the urgent need for infrastructure and policy interventions such as public parks, active transport promotion, and culturally sensitive fitness initiatives aligned with Vision 2030 health transformation strategies [4],[15].

### 4.6. Comparison with global evidence

When situated in the global context, the prevalence of obesity in Aseer (25.3%) is comparable to rates in high-income Western countries but exceeds those in many Asian and African nations [2],[9]. The convergence of dietary transition, sedentary lifestyles, and cultural constraints has accelerated the obesity epidemic in Saudi Arabia, echoing trends seen in other rapidly developing economies [11]. Unlike Western settings, however, the gender disparity is pronounced, underscoring the influence of sociocultural environment on obesity risk.

### 4.7. Policy and public health implications

The study findings underscore the need for targeted obesity prevention strategies in Aseer. Policies should focus on 1) nutrition education, addressing high consumption of sugary foods and promoting traditional diets rich in whole grains, legumes, and fresh produce, 2) promotion of physical activity, especially among women, through culturally tailored opportunities, 3) regional surveillance systems to capture subnational variations in obesity determinants and outcomes, and 4) integration into Vision 2030 strategies, ensuring alignment with broader national health promotion and chronic disease prevention goals [15],[22].

### 4.8. Strengths and limitations

The study's strengths include its focus on a regionally under-researched population, use of standardized tools (WHO STEPS), and robust multivariate analysis. However, limitations must be acknowledged. Because height and weight were self-reported, the true prevalence of obesity may be underestimated. Self-reported anthropometrics are known to be affected by over-reporting of height and under-reporting of weight, which can produce conservative BMI estimates. In addition, online recruitment may have overrepresented younger, urban, and more educated adults, while underrepresenting older or rural residents. The cross-sectional design precludes causal inference, and reliance on online recruitment may underrepresent older and rural populations. Future longitudinal studies and biomarker-based dietary assessments are recommended to strengthen the evidence base.

## 5. Conclusions

This study provides region-specific evidence on obesity determinants in Aseer and adds valuable data to Saudi obesity surveillance. Female gender, older age, single marital status, and physical inactivity were key predictors. While sugary food consumption showed a positive trend, it was not statistically significant in adjusted models. Tailored interventions promoting physical activity and healthier dietary behaviors are needed, particularly for high-risk groups.

## Data availability statement

The datasets generated and analyzed during the current study are available from the corresponding author upon reasonable request. Any shared data will be fully anonymized and dissociated from all identifying characteristics to comply with ethical and privacy requirements.

## Use of AI tools declaration

The authors declare they have not used Artificial Intelligence (AI) tools in the creation of this article.
